# Antimicrobial resistance in Chilean marine-farmed salmon: Improving food safety through One Health

**DOI:** 10.1016/j.onehlt.2021.100219

**Published:** 2021-01-24

**Authors:** Ivonne Lozano-Muñoz, Jurij Wacyk, Cristina Kretschmer, Yesseny Vásquez-Martínez, Marcelo Cortez-San Martin

**Affiliations:** a Laboratorio de Nutrición, Departamento de Producción Animal, Facultad de Ciencias Agronómicas, Universidad de Chile, Santa Rosa 11315, La Pintana, Santiago, Región Metropolitana CP 8820808, Chile; b Laboratorio de Virología Molecular y Control de Patógenos, Facultad de Química y Biología, Universidad de Santiago de Chile, Av. Libertador Bernardo O’higgins 3363, Santiago, Región Metropolitana CP 9170022, Chile; c Programa Centro de Investigación Biomédica Aplicada, Escuela de Medicina, Facultad de Ciencias Médicas, Universidad de Santiago de Chile, Av. Libertador Bernardo O’Higgins 3363, Santiago, Región Metropolitana CP 9170022, Chile

**Keywords:** Antimicrobial resistance, Food safety, Farmed salmon,, One Health,, Food chain

## Abstract

Aquaculture is seen as an essential requirement for improving food security and nutrition. Fish such as salmonids are a primary source of protein and essential nutrients. Aquaculture provide income for communities across the world and have a smaller carbon footprint than terrestrial animal-production systems. However, fish diseases are a constant threat, and the use of antibiotics is a source of concern due to its adverse impacts on the environment and human health. Chilean salmon farming has made several efforts to reduce the use of antibiotics for the eradication of piscirickettsiosis, a disease caused by the gram-negative bacteria *Piscirickettsia salmonis*. Excessive amounts of antibiotics continue to be used in Chilean aquaculture, playing an important role in the emerging public health crisis of antimicrobial resistance. Without doubt, *P. salmonis* is becoming increasingly resistant to important frontline antimicrobial classes, with severe implications for the future treatment of infectious human and animal diseases. Antimicrobial-resistant bacteria as well as antibiotic residues from salmon production are spreading in the environment, and thus both salmon food commodities and wild organisms can become a source of resistant bacteria that can be transmitted to humans as foodborne contaminants. This urgent threat needs to be addressed by implementing national strategies in compliance with international standards that include both prudent antimicrobial use in marine salmon farms and the investment towards a One Health approach, which combines human, animal and environmental health.

## Graphical abstract

**Figure:**
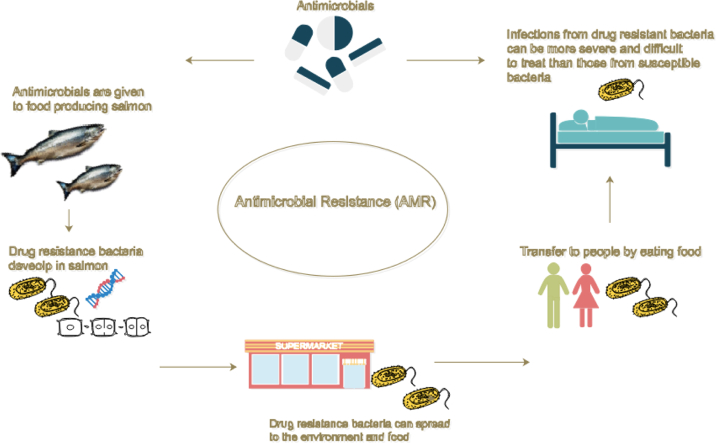

## 1 Introduction

Projections on population growth and consumption habits indicate that by 2050, animal protein consumption will increase by 75% [1]. Producing sustainable and healthy food with limited land, freshwater, and nutrient resources will be one of the greatest challenges [2]. Aquaculture may provide alternatives that can reduce our environmental footprint [2], provide health benefits [3], and play an important role in fulfilling food demand [1]. A great challenge for this industry is to meet this demand in a sustainable way [4]. Throughout the world, more than 300 species of fish are produced under controlled or semi-controlled conditions, with carp, tilapia, and salmonids being the most common in terms of production volume [5].

Salmon farming has grown exponentially, with 3.3 million tons produced in 2016 [2]. Chile is the second-largest producer of salmonids after Norway [6]. Due to its high economic and nutritional value, salmon production has become an integral part of the global aquaculture market, providing several benefits for both human and environmental health [7]. Salmon is an important source of essential nutrients, including selenium, the B vitamins [8], omega 3 fatty acids, and vitamin D [9]. In contrast to what has been reported for the consumption of processed and red meat [10], pescetarian diet has been linked to lower per capita greenhouse gas emissions and a reduction in the risk of developing coronary disease and metabolic diseases such as diabetes [7]. Compared to land animals, fish are also known for their lower feed conversion rate, requiring fewer natural resources per kilogram of protein produced [11].

Although aquaculture is generally considered a healthy and sustainable food source [12], some production practices (e.g. chemotherapy use) and environmental side-effects (e.g. heavy metal accumulation) might raise some environmental and health concerns [13]. For example, the development of antimicrobial resistance (AMR) and its associated environmental impacts [14,15] are a growing public concern which challenges aquaculture growth [12].

The excessive use of antibiotics in salmon farming has been questioned in Chile for over a decade [12,14,[16], [17], [18], [19], [20]]. Antibiotic overuse is a major problem that greatly impacts the environment and has resulted in multiple antimicrobials resistance bacteria [14,18]. The contribution of aquaculture to the emergence of AMR must be controlled, or new antimicrobials will not be sufficient to prevent major crisis in the treatment of bacterial infectious diseases in both animal and human populations [14]. In 2016, Norway used 201 kg [21] of antibiotics to produce 1.3 million tons of salmon (0.01 g/ton) [2]. That same year, Chile used 382,500 kg of antibiotics to produce 727,812 tons of salmon, and 334,100 kg of antibiotics were reported for 2019 (500 g/ton) [22], indicating the continuous excessive antibiotic use in Chilean salmon farming, which threatens to cause environmental and health issues. Antibiotic administering in salmon farming is mainly through the administration of medicated feeds, which results in antibiotics leaching into the environment [23]. In addition, undigested food and fish feces contain unabsorbed antibiotics, and antibiotic metabolites can remain in the water as sediment for an extensive period [12]. Some studies suggest that 70% to 80% of the antibiotics administered to fish are excreted into the water, which can alter microbial communities and sediment biodiversity, and contribute to the emergence of antimicrobial resistance bacteria [12]. Thus, before using antibiotics, ecotoxicity studies should be performed based on the international guidelines for environmental impact assessments for veterinary medicinal products [21,24,25].

The Aquaculture Stewardship Council (ASC) manages the world's salmon aquaculture sustainability standards, while the Global Salmon Initiative (GSI) was established by major salmon production companies around the world that share the vision of offering a healthy and sustainable source of protein to feed a growing population while minimizing their environmental footprint and improving their social contribution [26]. This initiative (i.e., GSI) has committed to having 100% of its member farms ASC-certified. In Norway, the state is collaborating with companies to increase the number of ASC-certified salmon farms. However, Chilean fish health policies are not well matched with the ASC's salmon standards, and Chilean regulations do not prevent the use of antibiotics listed as critical to human health by the World Health Organization (WHO) [2].

To make a contribution to minimizing the spread of AMR, the aim of this study was to analyze the causes of excessive antibiotic use in salmon farming in Chile and propose measures to reduce it and its environmental and public health effects.

## 2 Methods

We first compare the criteria for salmon production practices set by the Aquaculture Stewardship Council (ASC) with the regulatory framework in Chile on the subjects of disease management, antibiotic treatment, and antibiotic resistance. We then review the literature on *Piscirickettsia salmonis* regarding its ubiquity in freshwater and its effects on marine environments and fish health, the presence of piscirickettsiosis (salmonid rickettsial septicemia, SRS) in marine and farming environments, *P. salmonis*’s sensitivity to antibiotics and intestinal microbiota imbalances that may favor SRS colonization. Finally, we review the literature on FLO usage in salmon production, antimicrobial resistance, and its impact on food safety.

## 3 Integration of Chilean policies into AMR One Health strategies: A comparison of the criteria set by the Aquaculture Stewardship Council and the regulatory framework in Chile

During 2019, the major cause of reported mortality in Chilean salmon farming was infection, with piscirickettsiosis being the most prevalent in marine farms, responsible for 83.3% of the total deaths. Of the total amount of antimicrobials used that year, 96.5% was administered to combat this disease in the seawater phase [27].

Currently, the international standards for sustainable salmon farming are set by the ASC and the Global Aquaculture Alliance (GAA). The GAA certifies the Best Aquaculture Practices (BAP), which is a certification program that encompasses compliance with the Global Food Safety Initiative and the Global Sustainable Seafood Initiative [28]. The ASC manages one of the world´s leading sustainability standards for salmon, and the GSI is committed to having 100% of its member farms ASC-certified [2]. In Chile, the guidelines for salmon farming are issued by the SERNAPESCA government authority.

The total amount of antibiotics used in Chile in 2018 was 322.7 tons (to produce 923,900 tons of salmon), which represented a 17.8% decrease relative to 2017 (393.9 tons to produce 855, 326 tons of salmon). However, in 2019, 334.1 tons of antibiotics were used to produce 989,546 tons of salmon [22]. This is over 2000 times more than what Norway used, 0.201 tons of antibiotics to produce 1.3 million tons of farmed seafood, 95% of which was Atlantic salmon [2]. The maximum allowed dose of the antibiotic used in the aquaculture of freshwater reared-salmonids is 10–15 mg of FLO per kg of fish [29]. In 2017, Chile used around 49 times this regulatory dose [22].

There are important differences between the ASC [30] standards and government requirements in Chile [[31], [32], [33]]. Regarding disease management and the issue of mortality in salmon farming, the ASC standards establish that 100% of mortality events must receive a post-mortem analysis of a statistically relevant number of fish from the event. In the case of Chile, only fish without an apparent cause of mortality receive a laboratory diagnosis in relation to infectious disease. In Chilean salmon farming, the most prevalent infectious disease in the seawater phase continues to be SRS, and *P. salmonis*, the pathogen involved, is classified at a secondary level that includes mortality without apparent cause [31].

The ASC establishes a maximum unexplained mortality rate of ≤40% of total mortality for each of the two previous production cycles. Chile’s total mortality (excluding that caused by environmental and predatory causes) limit is ≥0.35% per week in one or more cages or >0.05% mortality in one or more cages for five consecutive days. Given a production cycle of 16 to 18 months, this is a range of ≤22.4% to 25.2% of total mortality, which is 18% lower than the ASC standard. If this level is exceeded, the Chilean government requires a treatment plan that involves harvest measures or the use of antibiotics (Table 1).

**Table 1:** Comparison of the standards for responsible disease management from the Aquaculture Stewardship Council (ASC) and the Chilean legal framework.

Indicator [30]	ASC	SERNAPESCA^a^
1. Evidence of a fish health management plan for the identification and monitoring of fish diseases, parasites, and environmental conditions relevant for good fish health, including implementing corrective action when required.	Required	Yes (1)
		The health management plan established by the government must be followed. Salmon farms may adopt additional measures to those imposed by the regulations such as for smolt quality, vaccine usage, immunostimulants, functional diets, transport conditions, and crop densities, among others. [32].
2. Site visits by a designated veterinarian at least four times a year, and by a fish health manager at least once a month.	Required	Partially (0.5)
		Every farm will be subject to two annual sanitary visits to confirm the absence of the high-risk diseases on List 1 (SRS is in List 2).
		Number of samples required: 5% of the fish should be extracted with a 95% confidence limit [33].
3. Percentage of dead fish removed and disposed of in a responsible manner.	100%	Yes (1)
		Although 100% mortality is not specified, there is reference to the daily withdrawal of mortalities with the exception of egg incubation centers, where removal is performed according to the company-established strategy [31].
4. Percentage of mortalities that are recorded, classified, and receive a post-mortem analysis. If the on-site diagnosis is inconclusive, this standard requires an off-site laboratory diagnosis. A qualified professional must conduct all diagnoses. All mortality events must receive a post-mortem analysis, not necessarily every fish. A statistically relevant number of fish from the mortality event must be analyzed.	100%	Not required (0)
		The daily mortality classification as a proportion of total dead fish is on two levels: Level I is only according to the external characteristics of the fish (10 categories); Level II is performed on fish classified as mortality without apparent cause.^b^ This secondary classification^c^ is performed based on the presence of anatomopathological signology characteristics of a disease, detected by specialized personnel at the culture center, a clinical diagnosis by a veterinary doctor, or by a laboratory diagnosis [31].
5. Maximum viral disease-related mortality on farm during the most recent production cycle. Viral disease-related mortality shall include unspecified and unexplained mortality, as it could be related to viral disease.	≦10%	Not required (0)
		Production cycle for *Salmo salar* is 16–18 months. No maximum mortality value due to viral disease has been established.
6. Maximum unexplained mortality rate from each of the previous two production cycles for farms with mortality.	≤40% of total mortalities	Partially (0.5)
		Maximum unexplained mortality: a value ≥0.35% weekly in one or more cages or 0.05% mortality in one or more cages that exceeds 0.05% daily for five consecutive days. The production cycle of 16-18 months for *Salmo salar* yields a standard of ≤22.4% to 25.2 % of total mortalities. Every farm with the these or higher amounts of mortality must present an action plan that includes measures such as harvest, total elimination, or pharmacological treatments [32].
7. A farm-specific mortality reduction program that includes defined annual targets for reducing overall and unexplained mortality.	Required	Yes (1)
		Within 48 hours, every center notified as an alert center must submit an action plan for reducing transmission of the pathogen into the environment; it must include an increase in the frequency of mortality withdrawal to at least twice a day and incorporate the extraction of dying fish, harvest or partial/total elimination, and pharmacological treatments [32].

a SERNAPESCA: Servicio Nacional de Pesca y Acuicultura (http://www.sernapesca.cl/).

b Those dead fish that, according to external observation, have the characteristics of healthy fish and those dead fish that, due to their external characteristics, are associated with infectious pathologies [31].

c Secondary-level mortality classification: 1) vibriosis, 2) NPV, 3) furunculosis, 4) BKD, 5) SRS, 6) ISA, 7) ICH, 8) flavobacteriosis, 9) yersinosis, 10) mycosis, 11) amebiasis, 12) jaundice, 13) francisellosis, 14) streptococcosis, and 15) other diseases [31].

There are also important differences between the ASC standards [30] and the requirements of the Chilean government regarding antibiotic treatment standards. Chile allows for the use of eight different types of antibiotics in aquaculture that are banned in other salmon-producing or importing countries [19], and no withholding periods have been established for these approved antibiotics. Antibiotics listed as being critically important for human medicine by the WHO [34] are also allowed in Chile [35]. Among the antibiotics allowed by Chilean regulations that are listed as critically important to human medicine are a) quinolones: oxolinic acid 80%, Litoflox, Bandrol, flumequine 80%, Flox-Fed, and Flumepren; b) macrolides: erythromycin 50% and 80%; and c) aminopenicillins: Amox-Feed (see Table 2).

**Table 2:** Antibiotic treatment standards of the Aquaculture Stewardship Council (ASC) and the Chilean legal framework.

Indicator [30]	ASC	SERNAPESCA^a^
1. On-farm documentation that includes, at a minimum, detailed information on all chemicals and therapeutants used during the most recent production cycle, the amounts used (including grams per ton of fish produced), the dates used, which group of fish were treated and against which diseases, proof of proper dosing, and all diseases and pathogens detected at the site.	Required	Partially (0.5)
		Farms must keep a record of the treatments performed, which must be reported on a monthly basis to the Service through the Aquaculture Control System (SIFA) [35].
2. Allowances for the use of therapeutic treatments, including antibiotics or chemicals that are banned^b^ in the primary salmon producing or importing countries.^c^	None	Yes (0)
		There are 12 different types of generic and 25 branded antimicrobials authorized for use in salmonids in Chile, in contrast to the United States, where only four antibiotics are approved [19].
3. Percentage of medication events that are prescribed by a veterinarian.	100%	Yes (1)
		The use of antimicrobial pharmaceutical products should be prescribed by a veterinarian [35]
4. Compliance with all withholding periods after treatments.	Required	Partially (0.5)
		There are no established withholding periods for approved antibiotics. A veterinarian must assign an adequate withholding period that considers the Maximum Limits of Residues of the market to which the product will be sent.
5. Allowances for the prophylactic use of antimicrobial treatments d.	None	Yes (1) [35]
6. Allowances for the use of antibiotics listed as critically important for human^e^medicine by the World Health Organization.	None	Yes (0)
		The following antibiotics listed by the WHO as critically important for human medicine are allowed: Quinolones: oxolinic acid 80%, Litoflox, Bandrol, flumequine 80%, Flox-Fed, and Flumepren; Macrolides: erythromycin 50% and 80%; Aminopenicillins: Amox-Feed [35]
7. Limit on the number of antibiotic treatments over the most recent production cycle.	≤3	No (0) [35]
8. If more than one antibiotic treatment is used in the most recent production cycle, it must be demonstrated that the antibiotic load^f^ is as least 15% less than that of the average of the two previous production cycles.	Required	Not required (0) [35].
9. Documents demonstrating the farm has provided the company or entity to which the farm or producing company is directly selling its product with a list of all therapeutants used in production.	Required	Not required (0) [35].

a SERNAPESCA: Servicio Nacional de Pesca y Acuicultura (http://www.sernapesca.cl/).

b “Banned” means proactively prohibited by a government entity due to concerns about the substance. A substance banned in any of the primary salmon-producing or -importing countries cannot be used in any salmon farm, regardless of the product’s country of production or country of destination.

c Norway, the United Kingdom, Canada, Chile, the United States, Japan, and France.

d A designated veterinarian must certify that a pathogen or disease is present before prescribing medication.

e The fifth edition (2017) of the WHO list of “Critically important antimicrobials for human medicine” is available at: http://apps.who.int/iris/bitstream/10665/255027/1/9789241512220-eng.pdf?ua=1.

f The antibiotic load is the sum of the total amount of active ingredients for the antibiotics used (kg).

The ASC standards for salmon farming establish a number of treatments over the most recent production cycle, with a limit of ≤3, and the requirement that if more than one antibiotic treatment is used in the most recent production cycle, it must be demonstrated that the antibiotic load is at least 15% less than that of the average for the two previous production cycles. The farm must also provide a document to its buyers that shows a list of all therapeutics used during production. None of these standards are required by Chilean regulations (Table 2).

The ASC standards for salmon-farming production establish controls for the prevention of antimicrobial resistance that include a bio-assay analysis to determine resistance, the use of alternative permitted treatments, and antibiotic rotation. The Chilean legal framework does not establish any of these controls (Table 3).

**Table 3:** Bacterial antibiotic resistance standards of the Aquaculture Stewardship Council (ASC) and Chilean legal framework.

Indicator [30]	ASC	SERNAPESCA^a^
1. Bio-assay analysis to determine resistance when two applications of a treatment have not produced the expected effect.	Required	Not required (0)
		To monitor the efficacy of antimicrobials, a report of treatment failures should be submitted as established in the specific health program for surveillance and control of SRS [35].
2. When bio-assay tests determine resistance is forming, the use of an alternative permitted treatment or an immediate harvest of all fish on the site.	Required	Not required (0) [35]
3. Specific rotation, providing the farm has >1 effective medicinal treatment products available; every third treatment must be with a different family of drugs.	Required	Not required (0) [35]

a SERNAPESCA: Servicio Nacional de Pesca y Acuicultura (http://www.sernapesca.cl/).

For each of the points included in the standards for disease management (Table 1), antibiotic treatments (Table 2), and bacterial antibiotic resistance (Table 3), we have assigned a value of 1 to those situations in which the Chilean legal framework agrees with the ASC standards, a value of 0.5 when there is partial agreement, and a value of 0 when the requirements established by the ASC are absent in the Chilean regulations. As shown in Table 4, the comparison between the Chilean regulations and the ASC sustainable salmon standards showed a total deficiency in the controls for antimicrobial resistance and important divergences in the disease management and antibiotic treatment standards.

**Table 4:** Comparison of the Aquaculture Stewardship Council’s sustainable salmon standards for disease management, antibiotic treatment, and antibiotic resistance and those of the Chilean legal framework. Degree of homology: red, no homology; yellow, partial homology; green, total homology.

Area	Sub-area	Indicator	Score^a^
Disease management	A1	1	57.14
	A2	0.5	
	A3	1	
	A4	0	
	A5	0	
	A6	0.5	
	A7	1	
Antibiotic treatment	B1	0.5	33.33
	B2	0	
	B3	1	
	B4	0.5	
	B5	1	
	B6	0	
	B7	0	
	B8	0	
	B9	0	
Antibiotic resistance	C1	0	0
	C2	0	
	C3	0	

I = indicator, N°I = number of indicators.

a Score = (∑I∕N°I) × 100.

## 4 *Piscirickettsia salmonis*: The bacteria behind the excessive use of florfenicol in farmed salmon production

The rickettsias (Alphaproteobacteria) are a group of obligate intracellular [36] microorganisms that generally cause severe disease in their hosts (i.e., warm-blooded animals), ranging from fevers to certain types of typhus [37]. The first rickettsial infection in fish was reported 80 years ago and affected *Tetrodon fahaka*, from the Nile River in Egypt. The analyzed tissues of these diseased fish were found to have a rickettsia-like organism (RLO) located intracellularly [37].

The RLO responsible for causing Salmon Rickettsial Syndrome (SRS) was subsequently named *P. salmonis* [38], later it was established the new family *Piscirickettsiaceae* (class Gammaproteobacteria, order Thiotrichales) that infects fish.

Given the high prevalence of *P. salmonis* in salmonids grown in Chile, Contreras-Lynch et al. [39]. conducted an in-depth study that included the capture of wild fish surrounding salmonid farming areas in southern Chile. The results of this work interestingly showed that several teleost species were positive for *P. salmonis* (EM-90). Additionally, the strains found in *S. capensis* and *S. australis* were related to the LF-89 strains, and both strains (EM-90 and LF-89) are found in high prevalence in Chilean salmonids [39].

These antecedents indicate *Piscirickettsias* are widely distributed in diverse aquatic environments (both freshwater and seawater) and are present in various teleost species. However, it is important to note that despite *P. salmonis* being prevalent in wild fish, no pathognomonic signs were observed in any of the captured specimens [39]. Previous studies have concluded that *P. salmonis* is likely an opportunistic environmental pathogen with low levels of virulence and pathogenicity and an endogenous pathobiont colonizing the normal salmonid microbiome [20].

This allows us to suggest that RLOs are a part of the normal microbiota of aquatic animals and to therefore propose as a hypothesis that it is an alteration in the balance of the bacterial population in fish that leads to the development of the pathology piscirickettsiosis [20]. In this way, a microbiome study conducted on the European common cuttlefish *Sepia officinalis* showed that the relative abundance of bacteria belonging to the *Piscirickettsiaceae* family in the gills of these animals was 96.09%, which suggests these bacteria had reached a symbiotic relationship in the colonized cuttlefish gills [40].

Disease outbreaks and peak mortality due to *P. salmonis* have increased in Chile in three cultivated species, *Salmo salar*, *Oncorhynchus kisutch*, and *Oncorhynchus mykiss* [36], as well as in fish in the freshwater-culture stage [41]. SRS outbreaks and isolated strains of *P. salmonis* were reported to affect coho salmon in the X (Los Lagos) region of Chile in the spring of 1998, and they affected Atlantic salmon and rainbow trout from the X and XI regions in 2012, with mortalities between 2.2% and 21.5% [42]. It is important to highlight that in the case of Chile, Otterlei et al. [42] reported observing two well-differentiated phylogenetic groups, one of which was represented by the LF-89 strain. As demonstrated by Manuel et al. [73] and Casanova et al. [74], we currently have two prototype strains in Chile belonging to two different phylogenetic groups, which correspond to the strains LF-89 and EM-90. In Europe, the *P. salmonis* strains described in Ireland also showed two phylogenetic groups, as did the Scottish strains [43]. *Piscirickettsia. salmonis* has also caused outbreaks in the world's leading salmonid-producing country (i.e., Norway), though with low mortalities in Atlantic salmon [44].

In Canada, *P. salmonis* has been of low importance. Outbreaks with moderate mortality (a peak of 0.22% daily mortality) were reported in the spring of 1996 in Atlantic salmon that had recently been transferred to the sea stage on the east coast [45]. A recent study conducted in British Columbia, where juvenile coho salmon were analyzed between 2008 and 2018 in both the wild and hatchery state, showed that *P. salmonis* was in few samples, with only a 0.2% prevalence in the 2650 analyzed specimens. With the exception of the bacterium *Candidatus Branchiomonas cysticola* (Betaproteobacteria), which has had its highest prevalence of 89.3% in coho salmon [46], diseases with a prevalence >10% are considered parasitic. A similar study conducted with wild sockeye salmon in the Fraser River in Canada between 2012 and 2013 found only one positive fish among the 2005 specimens analyzed, giving a prevalence of only 0.05% [47]. These results seem to suggest that *P. salmonis* might be present in most salmonid culture systems, being able to cause severe SRS conditions. However, the reason behind the high P. salmonis prevalences in Chile remains unexplained.

Undoubtedly, the literature indicates that *P. salmonis* is the causative agent of SRS, also known as piscirickettsiosis. Two independent works [48,49] have shown that inoculating coho salmon with isolated *P. salmonis* strains causes lethargy, anorexia, and a darkening of the fish’s skin. The fish were also grouped and located at the surface, showing disorientation and erratic swimming. The pathognomic signs included anemia, pale gills and livers (or yellowish nodules in the liver), as well as congestive kidney and spleen failure. Hemorrhages at the base of the fins and petechial hemorrhaging in pyloric caeca were observed [48,49]. The inoculated fish were capable of causing disease in sentinel or cohabiting fish, demonstrating the ability of this bacterium to transmit horizontally [48].

The *P. salmonis* infection is systemic, causing SRS by bacterial colonization of the liver, kidney, heart, spleen, skeletal muscle, intestine, brain, gonads, and gills [50]. Although many of the pathognomic signs of piscirickettsiosis described in salmonids were observed in the RLO-infected Atlantic salmon in Norway, there were differences, including multifocal gill hyperplasia, stomach dilation and inflammation, and intestinal necrosis. Hepatotoxic effects, which are probably caused by a toxin from the bacteria, have also been described [44].

## 5 Florfenicol usage and antimicrobial-resistant bacteria

Antibiotic contamination is one of the greatest public health challenges facing the human population worldwide [[51], [52], [53], [54]]. The health hazards involved in antibiotic usage in aquaculture include the development of antimicrobial resistance (AMR) and the presence of antimicrobial residues in the environment and aquacultural seafood products [53]. These food products can be consumed without having undergone prior processing and therefore present a substantial risk for the transfer of AMR to humans [53]. The way to prevent the presence of these contaminants is by implementing a food safety system that establishes controls on and the use of only approved drugs. The conditions for approval should specify the species for which the drug is approved for use, the disease or other circumstances for use, the dosage regimen, administration route and withdrawal period, and the maximum residue level (MRL) allowed [29]. Based on toxicological assessments, the MRL index estimates the amount of a substance in food that can be ingested over a lifetime by humans without significant health risks [55,56]. Although the MRL may be in compliance with regulations, the presence of these antibiotics may provide a selection and enrichment mechanism for resistant bacteria [12].

An approved antibiotic indicates that (a) the drug is safe and effective for a specific use in a specific animal species and that the food made from the treated fish is safe for people to eat; (b) the manufacturing process is adequate for preserving the drug´s identity, strength, quality, and purity; and (c) that the drug´s labeling is truthful and not misleading [57]. Banned antibiotics have been found in farmed fish [56,58] and fish meal, and high antimicrobial levels have been detected in the latter [58]. Therefore, farmed-fish consumption may involve risks for consumers, especially from its contribution to antibacterial resistance [12,56,58].

By 2019, 96.50% of the total antibiotics used in salmon farming in Chile were being used in the seawater phase, with Florfenicol (FLO) contributing 98.6% of this amount. Antibiotic use in Chile increased 3.5% from 2018 (322.7 g/ton) to 2019 (334.1 g/ton) [22], they are still overused, with amounts well above those of other salmon-producing countries (e.g., Norway, Scotland, and Canada). In Norway and Scotland, even farms that are not ASC-certified use fewer antibiotics, giving them the added advantage of cost savings. In 2016, Norway used 0.154 g/ton of fish, 95% of which was Atlantic salmon [2].

Unlike Norway, Chile has high mortality rates attributable to infectious diseases caused by microbes, particularly the intracellular pathogen *P. salmonis* that causes piscirickettsiosis, responsible for 50% to 97% of mortality from disease in the Chilean salmon industry (Rosas and Enríquez 2014). FLO is a synthetic broad-spectrum antibiotic whose structure is analogous to that of chloramphenicol and thiamphenicol. It is effective against both aerobic and anaerobic bacteria and is in the Amphenicol antimicrobial class, which is on the WHO’s list of highly important antimicrobials (antimicrobial classes highly important for human medicine)[34]. In certain geographic settings, this class may represent one of the limited therapies available for acute bacterial meningitis and respiratory infections [34]. Due to its side effects in the hematopoietic system and the possibility of bacterial resistance, FLO’s use is controlled in such places as the United States, the European Union, Japan, and Chile, with established maximum permitted residue levels in salmon [19].

Salmon infected by *P. salmonis* responds poorly or inconsistently to treatment with FLO [20]. One possible reason for this treatment failure is related to the intracellular location of the bacteria, where the antibiotic may not reach the concentrations necessary to kill or inhibit the pathogen. The effectiveness of FLO was first evaluated in *S. salar* against infection by *Aeromonas salmonicida*, studying doses of 5, 10, and 20 mg/kg BW/day for 10 days under laboratory conditions at 18.9 °C. No statistically significant difference was found in the efficiency of the three concentrations tested as measured by mortality [59].

To monitor the appropriate application of antibiotics to produce the desired effect while avoiding resistance, it is essential to standardize and validate protocols for assessing pathogen antibiotic susceptibility. This has been difficult to implement for *P. salmonis*. As a nutritionally demanding pathogen, the bacteria can grow slowly in enriched free cell media, but not in those recommended for sensitivity tests. A method for quantitatively studying the antibiotic resistance of *P. salmonis* was not reported until 2014 [60], which was then used by the CLSI to establish a recognized method for determining MICs for *P. salmonis* [61]. However, different factors can affect this determination such as the variable susceptibility shown by different isolates [62].

Another factor that can cause the poor efficiency of FLO treatments against SRS is the route of administration, which is through food. The studies that have defined the application doses and administration period were under laboratory conditions. These studies try to resemble the feeding conditions under field conditions, but they are not representative of the populations or feeding conditions undergoing an SRS outbreak and do not consider the changes in feeding patterns experienced by infected fish. The result is that the tissue antibiotic levels are lower than expected [63,64]. Another reason for the excessive use of antibiotics in Chilean aquaculture is the prophylactic administration of treatments before receiving a positive result on the causative agent and/or having isolated the pathogen for a disease diagnosis; the latter is essential for determining the MIC values [65].

The only route of administration approved for FLO is via medicated food, with feed as the sole ration for 10 consecutive days to result in a dose of 10–15 mg FLO per kg fish. Feed containing FLO should not be provided for longer than 10 days. The acceptable daily intake for total FLO residue is 10 μg/kg BW/day, while the microbiological acceptable daily intake established for FLO is 31 μg/kg BW/day or 1.9 mg/person/day [57].

If we consider the amount of antibiotics used in relation to salmonid production in Chile [22], the FLO dosage in salmon production in the seawater stage in Chile has exceeded that allowed for freshwater salmonid farming by almost 50 fold, yet a recent study [66] has established an even higher dosage regimen (20 mg FLO/kg BW for 15 days).

Antibiotic-resistant bacterial infections have spread widely throughout the world [[51], [52], [53]], and a large increase in the number of cases has been registered, as demonstrated by the increase in the mortality rates for infectious diseases [[67], [68], [69]]. The elevated use of FLO has resulted in selecting for multidrug-resistant bacteria [18,20,[70], [71], [72]] in the farmed Atlantic salmon’s intestinal microbiota, and their feces are an important vehicle for the dispersion of these resistant bacteria and antibiotic-resistance genes [18].

Several studies in Chile have reported that *P. salmonis* has developed a resistance to antibiotics [18,71,72]. The presence of plasmids, integrons, bacteriophages, and DNA sequences involved in horizontal gene transfer and high-frequency DNA recombination processes in *P. salmonis* suggests the use of antimicrobials in salmon aquaculture and presence of antimicrobial residue in the environment is creating a critical point that is generating and disseminating new antimicrobial-resistance combinations among bacterial populations, with potentially negative effects on fish farming and human health [20].

## 6 Conclusions

As described by the ASC standards aimed at sustainable aquaculture, the amount of mortality associated with an efficient aquaculture system is a maximum of 40% unexplained mortalities out of the total mortalities from each of the previous two production cycles. In contrast, Chilean regulations set a lower mortality rate, with a maximum of 25.2% of total mortalities, which then requires an action plan involving the use of antibiotics. The ASC standards require that all (100%) mortality events receive a post-mortem analysis, whereas Chile only requires that mortality without apparent cause receive a laboratory diagnosis in relation to infectious disease. Aquaculture policies in Chile do not consider the use of antimicrobial-resistance controls in salmonid farms. Urgent changes are needed in Chilean regulations for the adequate control of SRS without pressuring the industry to use antibiotics.

Rickettsia-like organisms have been shown to be geographically ubiquitous and present in various aquatic species in both freshwater and marine environments. Whether in the wild or under farming conditions, they have been shown to be capable of causing disease, although they have also recently been described as being a part of the microbiota. The excessive use of antibiotics and high prevalence of SRS in Chile suggest that the cause of this disease is the indiscriminate use of antibiotics. However, further studies are required to obtain a better understanding of RLOs relative to the health balance in salmonids, especially given the enormous economic losses from piscirickettsiosis (SRS) and the excessive use of antibiotics that have been applied to control for the causative pathogen.

We do not currently understand the susceptibilities of the variants that cause outbreaks, therefore, the current treatments are not necessarily effective against *P. salmonis*. More studies are also needed to develop a better understanding of the mechanisms involved in the antibiotic resistance of *P. salmonis*, which would contribute to reducing the use of antibiotics in aquaculture.

Florfenicol is badly abused in the production of salmonids in Chile. This antibiotic, used to treat SRS, is on the WHO’s list of those antibiotics that are highly important for human medicine [34]. The use of FLO in one of the primary Chilean salmon-importing countries is approved in salmon farming for the control of mortality in freshwater-reared salmonids. However, complete seawater-reared salmonids studies are needed, including (a) toxicology studies supporting human food safety, including the effects on human intestinal flora; (b) a release assessment describing the probability that the proposed new dosage and its use will not result in the dissemination of resistant bacteria; (c) a consequence assessment describing the potential human health consequences of exposure to the defined resistant bacteria; and (d) an environmental assessment that examines the potential environmental impacts of FLO in the receiving waters as a result of its use. These studies must be performed prior to the continued use of this antibiotic in Chile.

## Author contributions

All authors contributed to the study conception and design. All authors performed the literature searches, data analyses, and writing. All authors commented on previous versions and critically revised the work.

## Declaration of Competing Interest

The authors declare that they have no conflict of interest.
